# Differential Recruitment of Pre-mRNA Splicing Factors to Alternatively Spliced Transcripts In Vivo

**DOI:** 10.1371/journal.pbio.0030374

**Published:** 2005-10-25

**Authors:** Stephen A Mabon, Tom Misteli

**Affiliations:** **1**National Cancer Institute, National Institutes of Health, Bethesda, Maryland, United States of America; University of WisconsinUnited States of America

## Abstract

Alternative splicing in mammalian cells has been suggested to be largely controlled by combinatorial binding of basal splicing factors to pre-mRNA templates. This model predicts that distinct sets of pre-mRNA splicing factors are associated with alternatively spliced transcripts. However, no experimental evidence for differential recruitment of splicing factors to transcripts with distinct splicing fates is available. Here we have used quantitative single-cell imaging to test this key prediction in vivo. We show that distinct combinations of splicing factors are recruited to sites of alternatively spliced transcripts in intact cells. While a subset of serine/arginine protein splicing factors, including SF2/ASF, SC35, and SRp20, is efficiently recruited to the *tau* gene when exon 10 is included, these factors are less frequently associated with *tau* transcription sites when exon 10 is excluded. In contrast, the frequency of recruitment of several other splicing factors is independent of splicing outcome. Mutation analysis of SF2/ASF shows that both protein–protein as well as protein–RNA interactions are required for differential recruitment. The differential behavior of the various splicing factors provides the basis for combinatorial occupancy at pre-mRNAs. These observations represent the first in vivo evidence for differential association of pre-mRNA splicing factors with alternatively spliced transcripts. They confirm a key prediction of a stochastic model of alternative splicing, in which distinct combinatorial sets of generic pre-mRNA splicing factors contribute to splicing outcome.

## Abstract 2

Quantitative single-cell imaging reveals distinct combinations of splicing factors recruited to sites of alternatively spliced transcripts in intact cells.

## Introduction

Pre-mRNA splicing is one of the key steps in the maturation of newly synthesized transcripts. During the splicing process, non-coding intron sequences are removed and the coding exon sequences are joined in a multi-step reaction, carried out by the spliceosome, a large protein complex consisting of small ribonucleoprotein particles (snRNP), and associated non-snRNP proteins [1,2]. The major class of non-snRNPs are the serine-arginine rich SR-proteins, characterized by the presence of one or two RNA-binding domains and a C-terminal SR-rich domain (RS-domain)[3,4]. The spliceosome carries out the splicing reaction in a series of complex spatial and temporal conformational rearrangements leading to the alignment of the reaction substrates and formation of the spliced mRNA [1,2].

While many introns are constitutively excised during splicing, other introns are subject to alternative splicing. During the alternative splicing reaction multiple mRNA species are generated by combinatorial inclusion or exclusion of exons [5–9]. Alternative splicing is rapidly emerging as a ubiquitous cellular event with estimates suggesting that 60%–90% of human genes are alternatively spliced [10]. Alternative splicing thus contributes significantly to protein diversity beyond that encoded in the genome sequence. Defects in alternative splicing and generation of aberrant ratios of multiple mRNA isoforms from a single gene are now also recognized as major contributors to human disease [11–13]. Amongst many others, alternative splicing defects have been identified as the cause of numerous diseases including β-thalassemia, cystic fibrosis, and premature aging [11].

The molecular basis for how alternative splice sites are selected is still largely unclear [7]. Although a handful of, often tissue-specific, dedicated splice site selection factors have been identified [13–16], the predominant model for splice site choice envisions that selective usage of an exon is due to the differential association of distinct sets of generic splicing factors [8,17]. This model is indirectly supported by the observation that in vivo and in vitro titration of multiple factors against each other modulates alternative splice site switching, presumably by antagonistic competition for splice sites [18–21]. A key prediction from this model is that alternatively spliced substrates should have different sets of splicing factors associated with them. Biochemical approaches to isolate spliceosomes with differential splicing factor composition have so far been unsuccessful, likely due to the heterogeneity of in vitro reactions and/or the transient nature of the association of splicing factors with their templates [2].

To circumvent these methodological limitations, we applied quantitative single cell microscopy to ask whether differential sets of splicing factors associate with alternatively spliced transcripts. This approach is based on the fact that pre-mRNA splicing factors are non-randomly distributed within the mammalian cell nucleus. The vast majority of pre-mRNA splicing components are enriched in distinct nuclear sub-compartments termed speckles or splicing factor compartments [22,23]. Despite the local concentration of splicing factors in these nuclear domains, they are most likely not active sites of pre-mRNA splicing but represent storage/assembly sites for splicing components [24–26]. Activation of genes near splicing factor compartments results in the physical recruitment of splicing factors from speckles and their accumulation at sites of active transcription and splicing [24]. This accumulation can be detected by quantitative fluorescence microscopy and used as an in vivo assay for recruitment of factors to pre-mRNA [26–28]. The observed recruitment is dependent on the functional interaction of splicing factors with the pre-mRNA since transcripts with no introns or mutant pre-mRNAs that cannot be spliced do not recruit splicing factors [28]. In addition, mutant splicing factors do not accumulate efficiently at transcription sites [27]. Using this quantitative in vivo single cell microscopy assay, we here provide evidence for differential recruitment of several splicing factors to alternatively spliced transcripts. Our findings confirm a key prediction of a stochastic, combinatorial recruitment model for alternative splicing.

## Results

### An In Vivo Assay for Recruitment of Splicing Factors to Alternatively Spliced Transcripts

We sought to establish an experimental system to study the recruitment of splicing factors to alternatively spliced transcripts in vivo. As a model system we used the *tau* gene, which encodes a microtubule binding protein and whose aberrant alternative splicing is the cause of frontotemporal dementia and parkinsonism linked to chromosome 17 (FTDP-17), a parkinsonism-like neurological disorder [29]. In healthy individuals, exon 10 of the *tau* gene is included or excluded from the mRNA with roughly equal probability during pre-mRNA splicing [29]. In contrast, among patients with FTDP-17 mutations near the 5′ splice site of exon 10 result in predominant inclusion of exon 10 [29–31]. A minigene system consisting of *tau* exon 10 flanked by partial *tau* intron sequences and *HIV TAT* exons has previously been used to mimic the alternative splicing behavior of the full-length wild-type (wt) and mutant *tau* gene in COS-7 cells (Figure 1A) [30,31].

**Figure 1:**
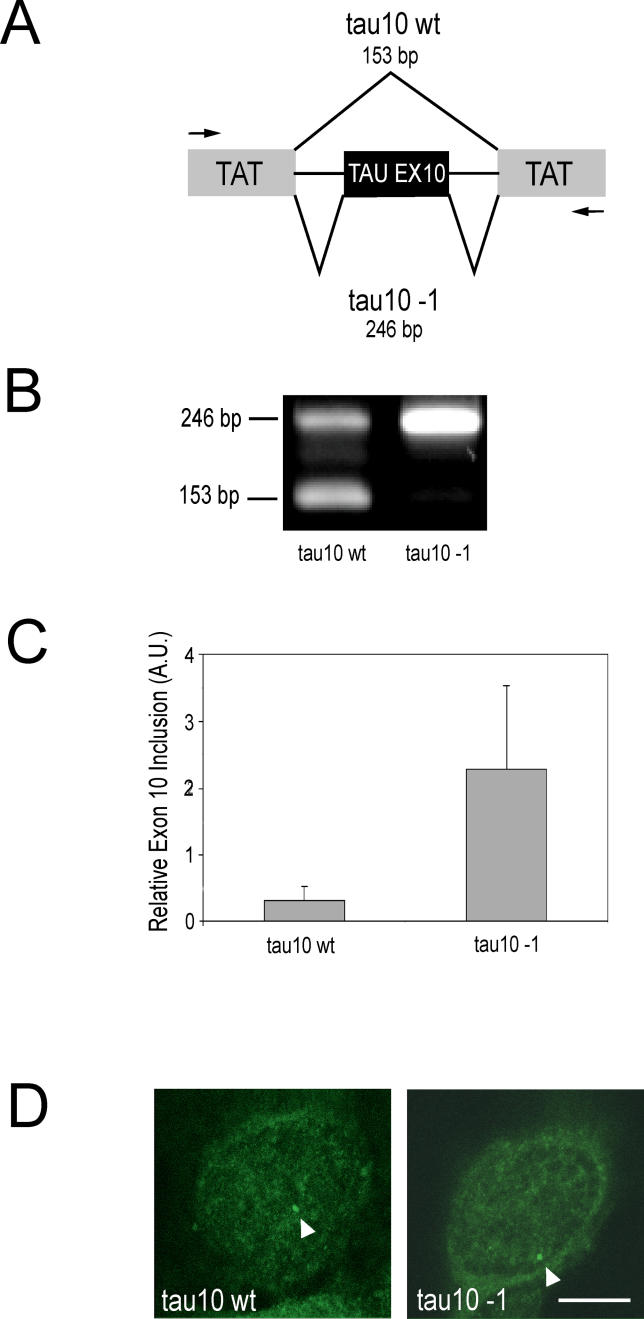
Characterization of Stable Cell Lines Expressing Alternatively Spliced *tau* Minigenes (A) Schematic representation of the *tau* exon 10 minigene system. Alternative inclusion or exclusion of exon 10 as observed for endogenous *tau* is recapitulated in a minigene containing flanking *HIV-TAT* exons. (B) Preferential inclusion of *tau* exon 10 detected by gel electrophoresis. The wt*tau* exon 10 (tau10wt) is evenly included or excluded from the mRNA, whereas a *tau* exon 10 containing a T>C mutation at position −1 of exon 10 (tau10−1) is predominantly included in the same context. Exon 10 inclusion is detected as a 246-bp by RT-PCR using primers (arrows) in the TAT exons. Exon 10 exclusion is detected by a 153-bp band. (C) Quantitative real-time PCR analysis to determine exon 10 inclusion. Results are averages ± standard deviations from at least three experiments. (D) RNA-FISH using nick-translated probes against the *tau* minigene to detect sites of *tau* transcription. The vast majority of cells in a stable cell population contained one or two *tau* minigene insertion sites. Arrowheads indicate *tau* transcription sites. Scale bar = 3.5 μm.

We used this well-characterized minigene to establish an experimental system to study potential differences in recruitment of splicing factors to alternatively spliced transcripts in vivo. To this end, monoclonal stable COS-1 cell lines expressing either tau10wt or tau10−1 were generated (see Materials and Methods for details). In COS-1 cells stably expressing tau10wt, exon 10 was predominantly excluded, whereas in tau10−1 stable cell lines, exon 10 was predominantly included (Figure 1B). This pattern is identical to that observed for endogenous tau in patients and in the transiently expressed minigene system [29,30]. Quantitation by real-time PCR analysis demonstrated a ∼ 5-fold increase in exon 10-containing mRNA in tau10−1 cells compared to tau10wt cells (Figure 1C). The transcription site of the stably integrated minigene was readily detectable by RNA fluorescence in situ hybridization (FISH) using nick-translated probes to full-length *tau* mRNA (Figure 1D). Cell clones for both tau10wt and tau10−1 were isolated containing the transgene in a single genomic integration site (Figure 1D).

### Differential Recruitment of SR-Proteins to Alternatively Spliced Transcripts

We used this experimental system to ask whether splicing factors associate differentially with alternatively spliced transcripts in vivo. To this end we took advantage of a previously characterized quantitative single cells splicing factor recruitment assay [27]. In this assay recruitment of a splicing factor to a specific transcription site is detected by simultaneous FISH to visualize the site of transcription and indirect immunofluorescence (IF) using specific antibodies to detect endogenous pre-mRNA splicing factors. As previously described, positive recruitment of splicing factors is defined as > 2-fold accumulation of splicing factor signal at the site of transcription above the global nucleoplasmic signal as determined by quantitative linescan analysis of single transcription sites (Figure 2A; see Materials and Methods for details) [27]. The obligatory U2 associated factor U2-B′′ served as a positive control and to determine the detection sensitivity of the recruitment assay. As expected U2-B′′ was detected at sites of transcription with high frequency both in cells expressing *tau* exon 10wt or exon 10−1 (Figures 2A and 2 B). Recruitment was detected at the site of *tau* transcription in 78 ± 6% of exon 10wt cells and 77 ± 14% of exon 10−1 cells (Figure 2A and 2B). Somewhat lower accumulations of typically between 1.3–1.9 fold over the nucleoplasmic signal were found in most other cells, and no *tau* transcription sites were detected that were entirely devoid of U2-B” signal (unpublished data). In contrast to the obligatory splicing factor U2-B”, the SR protein SF2/ASF showed differential recruitment between tau10wt and tau10−1 cells (Figure 2A and 2B). Endogenous SF2/ASF was recruited to transcription sites in more than 68 ± 6% of tau10−1 cells, but accumulated at transcription sites in only 31 ± 2% of tau10wt cells (Figure 2B). This difference in accumulation was significant at the *p* < 0.005 level in a Student *t*-test. The SR protein SC35 behaved differently from SF2/ASF in that it was only poorly recruited to either transcript (Figures 2A and 2B). SC35 was recruited to *tau* transcription sites in 38 ± 13% of tau10wt cells, and to 48 ± 2% of sites in tau10−1 cells (Figure 2B). SRp40 in contrast was recruited to the *tau* transcript in more than 80% of cells, regardless of the nature of the transcript (Figure 2B). Similar observations were made in several clonal cell lines (unpublished data). The observations that SF2/ASF was differentially recruited to the two *tau* transcripts, while SC35 was recruited to neither and SRp40 was recruited to both indicate that distinct SR proteins are differentially recruited to sites of alternatively spliced transcripts in vivo.

**Figure 2:**
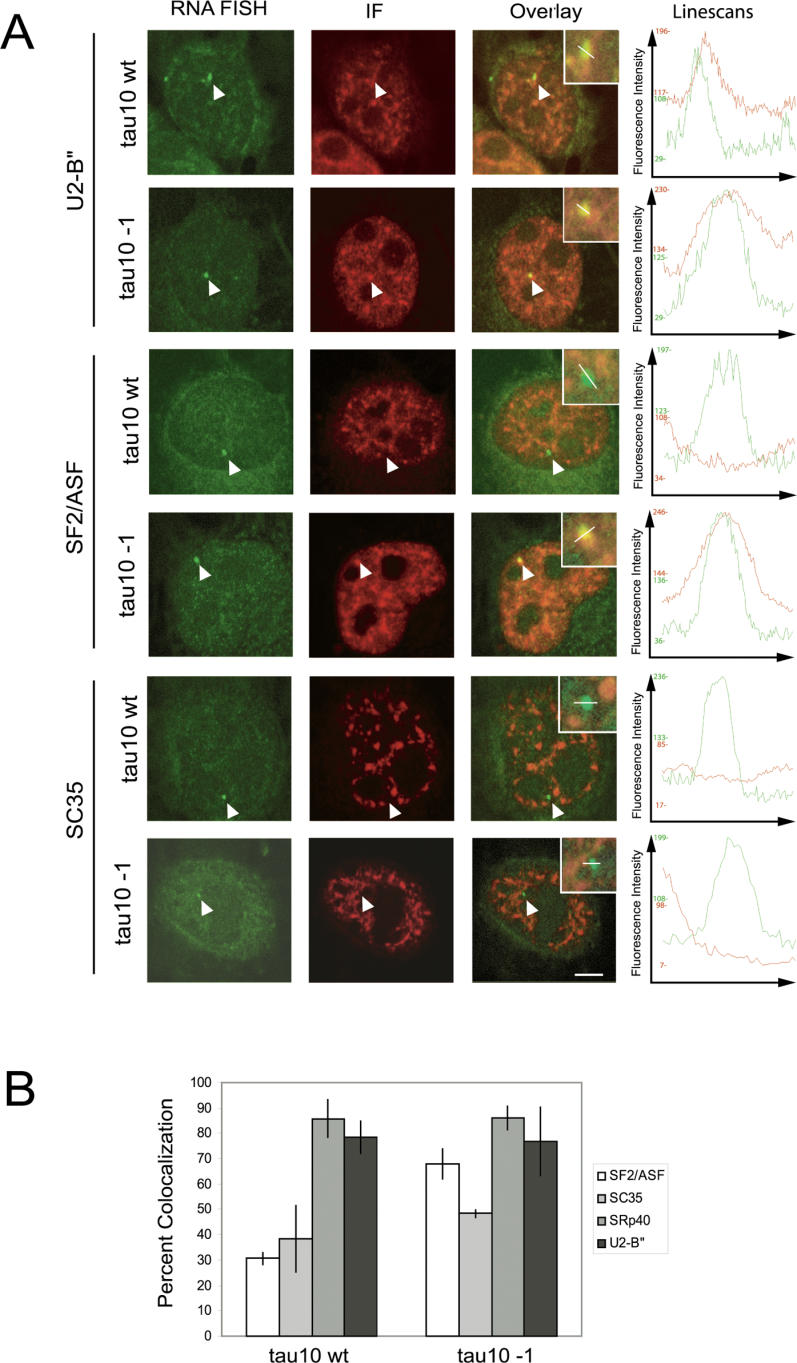
Differential Recruitment of Endogenous Pre-RNA Splicing Factors to Alternatively Spliced Transcripts (A) Recruitment of splicing factors to *tau* transcription sites detected by combined RNA-FISH using specific probes against the *tau* minigene (green) and IF microscopy with specific anti-splicing factor antibodies (red). Arrowheads indicate *tau* transcription sites. All associations were confirmed by linescan analysis. Scale bar = 2.5 μm. (Inset: higher magnifications of the transcription site) Lines indicate the location of the linescans. (B) Quantitation of percentage of cells with colocalization of *tau* RNA-FISH and splicing factor signals. Values represent averages from at least 100 transcription sites from at least three experiments ± SEM.

To further validate the correlation between recruitment and exon 10 inclusion, we used a FISH probe complementary to exon 10, thus specifically detecting mRNA containing exon 10 (Figure 3). As expected, splicing factors accumulated at sites of transcription of *tau* exon 10-containing pre-mRNA (Figure 3). This probe allowed us to test two predictions of differential recruitment. If the extent of recruitment indeed is related to splicing outcome, splicing factors should also be recruited to the subpopulation of transcription sites in tau10wt cells, which generate detectable levels of exon 10-containing *tau* pre-mRNA. To test this prediction we used the exon10-specific FISH probe to visualize tau10wt cells, which generate detectable levels of exon 10-containing mRNA and determined whether SF2/ASF and SC35 are recruited to those sites (Figure 3). As predicted, SF2/ASF and SC35 were recruited to the subpopulation of transcription sites in wt cells, at which exon 10-containing RNA could be detected (Figure 3A). Although fewer than 30% of all transcription sites in wt cells had splicing factors associated with them when visualized with a full-length *tau* probe, more than 90% of the subpopulation of wt cells in which significant levels of exon 10-containing RNA were detected with the exon 10-specific probe recruited SF2/ASF and SC35 (Figure 3B). As expected, no increase in the percentage of splicing factor recruitment was found in the tau10−1 cell line when the exon 10-specific probe was used (Figure 3B). This observation suggests that the recruitment behavior of splicing factors is primarily influenced by the predominant splicing outcome at a transcription site.

**Figure 3:**
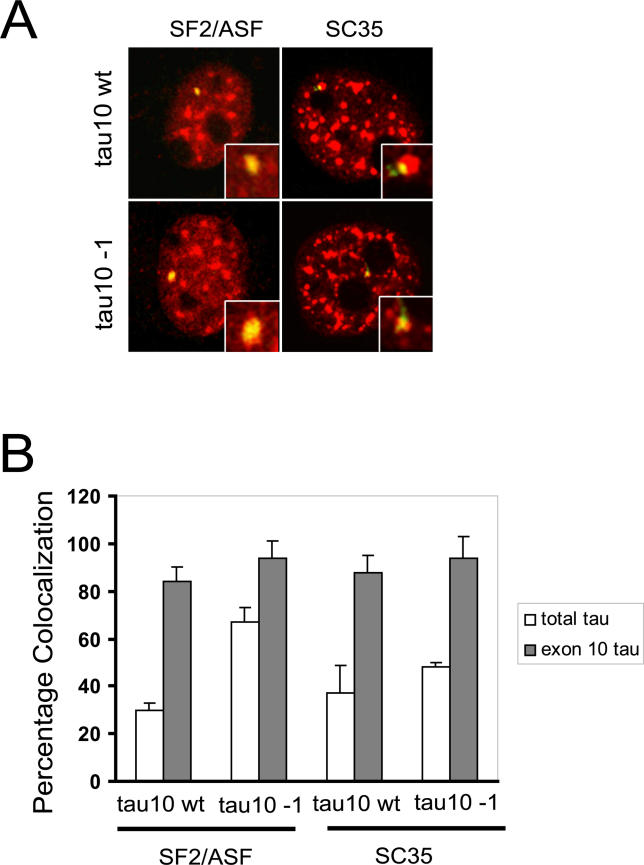
Recruitment of Endogenous Pre-RNA Splicing Factors to Transcription Sites Containing *tau* Exon 10 mRNA (A) Recruitment of splicing factors to *tau* transcription sites detected by combined RNA-FISH using a specific probe against *tau* exon 10 (green) and IF microscopy with specific anti-splicing factor antibodies (red). All associations were confirmed by linescan analysis. Scale bar = 2.5 μm. (B) Quantitation of percentage of cells with colocalization of *tau* RNA-FISH and splicing factor signals. Values represent averages from at least 100 transcription sites from at least three experiments ± SEM. Recruitment to the subpopulation of transcription sites containing predominantly *tau* exon10 mRNA in tau10wt cells was as efficient as recruitment in tau10−1 cells.

A second prediction from a recruitment model is that the percentage of cells that generate detectable amounts of mRNA containing exon 10 should be similar to the percentage of cells in which recruitment of SF2/ASF and SC35 is detected when using the full-length probe. Using the exon 10-specific FISH probe, we indeed found that the percentage of cells containing an mRNA-FISH signal for exon 10 was virtually identical to the extent of recruitment observed using a *tau* full-length probe. In tau10wt cells, 38 ± 8% of cells were positive for exon 10, comparable to the 31 ± 2 % and 38 ± 13% of cells with recruitment of SF2/ASF and SC35, respectively. These observations demonstrate that recruitment of splicing factors is a reflection of the production of exon10-containing RNA at a site of transcription.

### Recruitment Is Determined by Splicing Outcome

Tau10wt and the tau10 −1 mutant differ in a single residue at position −1 in the 5′ splice site [30]. Although sequence analysis using manual approaches and EsEFINDER (http://www.rulai.cshl.org/tools/ESE) did not reveal any strong consensus binding sites for SF2/ASF or SC35 in this region (unpublished data), we asked whether the specific mutation in −1 was responsible for the differential association of SF2/ASF or whether differential recruitment of this factor was correlated with splicing outcome per se. To this end, we generated stable cell lines expressing *tau* minigenes containing a G >A mutation at position +3 (*tau*10+3) or a C > U mutation at position +14 (*tau*10+14). These mutations have previously been shown to result in preferential inclusion of exon 10, similar to tau10−1 in patient cells and in transient transfection assays [30]. As expected, stable cell lines expressing either tau10+3 or tau10+14 preferentially included exon 10 (Figure 4A). Quantitative real-time PCR analysis indicated that inclusion in these cell lines was 3- to 4-fold above that observed in tau10wt cells (Figure 4B). Similar to tau10wt and tau10−1 cell lines, typically a single integration site was detected in these cells by RNA-FISH (unpublished data). As observed for tau10−1, U2-B” and SRp40 showed no differential recruitment and these proteins were equally efficiently recruited to tau10wt as to tau10+3 or tau10+14 transcripts, with more than 80% of cells showing an accumulation in either cell type (Figure 4C). Similar to the situation in tau10−1 cells, SF2/ASF was strongly recruited to transcription sites in tau10+3 and tau10+14 cells, with more than 80% of cells showing co-localization (Figure 4C), but was only recruited to 31 ± 2% of transcription sites in tau10wt cells (Figure 4C). SC35 was recruited to 38 ± 5% of tau10wt transcription sites, but to more than 90% of transcription sites in tau10+3 or tau10+14. We conclude that recruitment of these proteins is determined by the splicing outcome and not by the nature of the mutation near the splice site. Taken together, these results suggest that SF2/ASF and SC35 are differentially recruited to alternatively spliced transcripts and that this recruitment correlates with splicing outcome.

**Figure 4:**
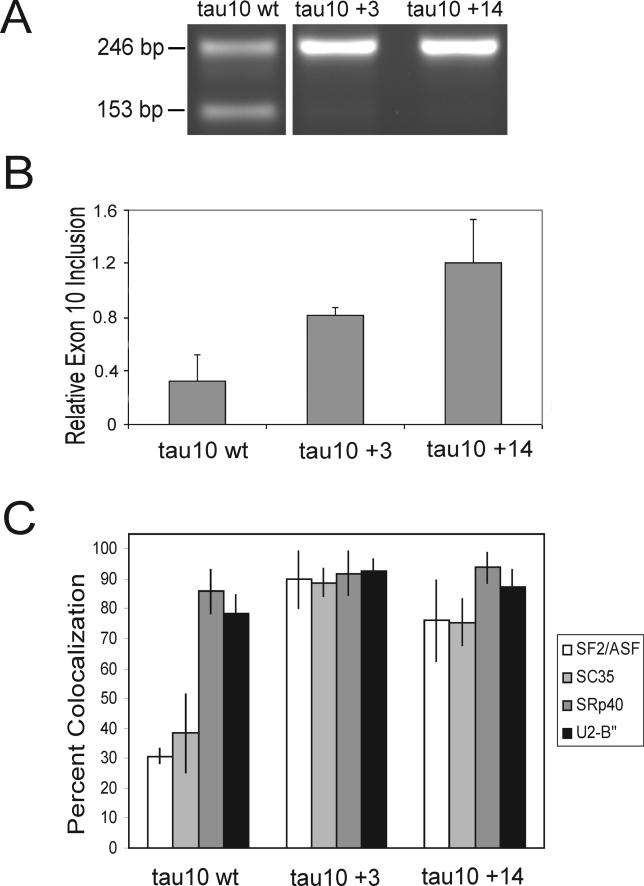
Mutation-Independent Differential Recruitment of Endogenous Pre-RNA Splicing Factors (A) Stable cell lines expressing minigenes containing a G>A mutation at +3 (tau10+3) or a C>U mutation at +14 (tau10+14). Both mutations give preferential inclusion of exon 10 as previously reported for transient expression. (B) Quantitative real-time PCR analysis demonstrating the preferential inclusion of exon 10 in tau10+3 and tau10+14 compared to tau10wt. Values represent averages ± standard deviations from at least three experiments. Values of included exon 10 are normalized to total minigene RNA. (C) Quantitative analysis of recruitment of endogenous splicing factors to tau10+3 or tau10+14. Recruitment of splicing factors to *tau* transcription sites detected by combined RNA-FISH using specific probes against the *tau* minigene and IF microscopy with specific anti-splicing factor antibodies. Percentage of cells with colocalization of *tau* RNA-FISH and splicing factor signals is indicated. Values represent averages from at least 100 transcription sites from three experiments ± SEM.

### Recruitment of Exogenous SR Proteins

The analysis of endogenous splicing factor recruitment is restricted by the availability of only a limited set of specific antibodies to SR proteins and simultaneous detection of multiple splicing factors is prevented by species cross-reactivity of the available antibodies. To expand our analysis we introduced epitope-tagged SR proteins into *tau*-expressing cells using transient transfection. Since transient transfection of SR proteins has previously been shown to be able to affect alternative splicing outcome [19], we first determined in a pilot experiment whether expression of SR proteins alters alternative splice site choice in the stably expressed *tau* minigene. Upon transfection of characterized T7-tagged SRp20, SRp30s, SRp55, 9G8, or SF2/ASF [32,33], a modest increase in inclusion was observed in tau10wt cells and no effect was evident in tau10−1 cells (Figure 5A). The absence of an effect was not due to sub-optimal transfection efficiency since a majority of cells expressed the T7-tagged SR proteins as confirmed by fluorescence microscopy staining (unpublished data). When analyzed in the recruitment assay, as a control and as observed for endogenous SF2/ASF, transiently transfected T7-SF2/ASF was preferentially recruited to tau10−1 (77 ± 5%) compared to tau10wt transcripts (54 ± 9%; Figure 4B), further confirming the transient expression approach. The percentage of recruitment-positive tau10wt cells appeared somewhat higher upon transient transfection compared to that observed for the endogenous protein (54 ± 9% for transient; 31 ± 2% for endogenous), most likely due to the generally more diffuse distribution of the overexpressed splicing factor [32]. Exogenous SRp20 behaved similarly to SF2/ASF and was differentially recruited in tau10−1 cells compared to tau10wt cells (Figure 5B). While T7-SRp20 was recruited in 56 ± 9% of tau10wt cells, the protein was found at tau-1 transcription sites in 81 ± 4% of cells (*p* < 0.005). No statistically significant differences were observed for the recruitment of 9G8, SRp30s and SRp55 with all proteins being recruited to more than 70% in either tau10wt or tau10−1 cells (Figure 5B). These observations suggest that SRp20 is similarly differentially recruited to alternatively spliced *tau* as SF2/ASF.

**Figure 5:**
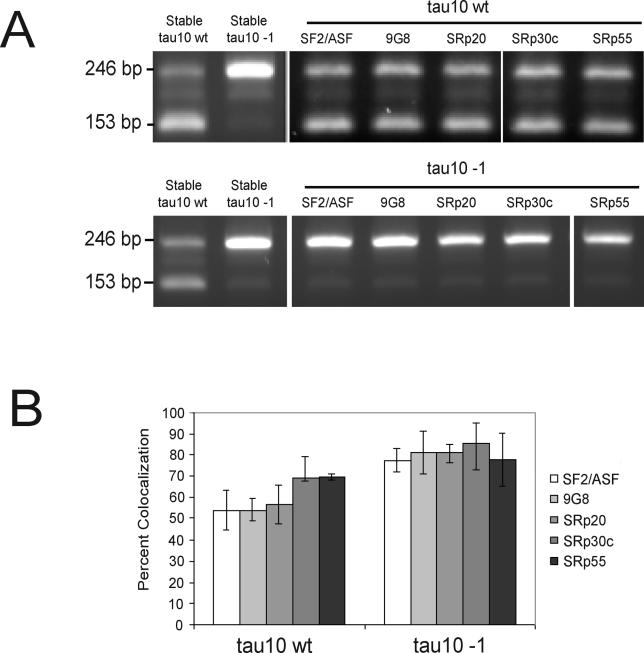
Differential Recruitment of Exogenous Pre-RNA Splicing Factors to *tau* Minigenes (A) Semi-quantitative RT-PCR analysis of tau alternative splicing upon expression of T7-tagged SR proteins in COS-7 cells expressing either tau10wt or tau10−1. Transient transfection of splicing factors does not affect the alternative splicing pattern of the tau minigene in stable cell lines. (B) Quantitative analysis of recruitment of transfected SR proteins to tau10wt or tau10−1. Recruitment of splicing factors to *tau* transcription sites detected by combined RNA-FISH using specific probes against the *tau* minigene and IF microscopy with anti-T7 antibody. Percentage of cells with colocalization of *tau* RNA-FISH and splicing factor signals is indicated. Values represent averages from at least 100 transcription sites from three experiments ± SEM.

### Mapping of Differential Recruitment Domains in SF2/ASF

Our results on endogenous and exogenous SF2/ASF demonstrate that this protein is preferentially recruited to the *tau* minigene when exon 10 is included. We sought to determine what parts of SF2/ASF were responsible for recruitment to tau10−1. SF2/ASF contains two RNA-binding domains in its N-terminal half and a characteristic SR-rich protein–protein interaction domain at its C-terminus (Figure 6A). To ask whether recruitment involved RNA and/or protein–protein interactions, we expressed a set of previously characterized domain deletion mutations of SF2/ASF in tau10−1 cells [32] (Figure 6A). As observed for the wt SF2/ASF protein, expression of the deletion mutants had no significant effect on splicing outcome of tau10−1 (Figure 6B). When the ability of these proteins to be recruited to tau10−1 was assessed, we found that deletion of any one domain resulted in reduction of recruitment from 48 ± 10% for the wt control to ∼ 30–40% for any of the deletion mutants, suggesting that no single domain alone is responsible for recruitment (Figure 6C and 6 D). In contrast, deletion of any combination of two domains resulted in almost complete loss of recruitment (Figure 6C and 6D). None of these latter mutants were recruited to transcription sites in more than 15% of cells (Figure 6C and 6D). Note that expression of the RS-domain alone results in cell toxicity and was not analyzed [32]. These observations suggest that all domains contribute to recruitment of SF2/ASF to tau10−1 and that both RNA binding as well as protein–protein interactions are required for efficient recruitment to alternatively spliced *tau*. The involvement of multiple domains in the efficient recruitment to alternatively spliced transcripts is similar to the requirements for recruitment to constitutively spliced transcripts [27].

**Figure 6:**
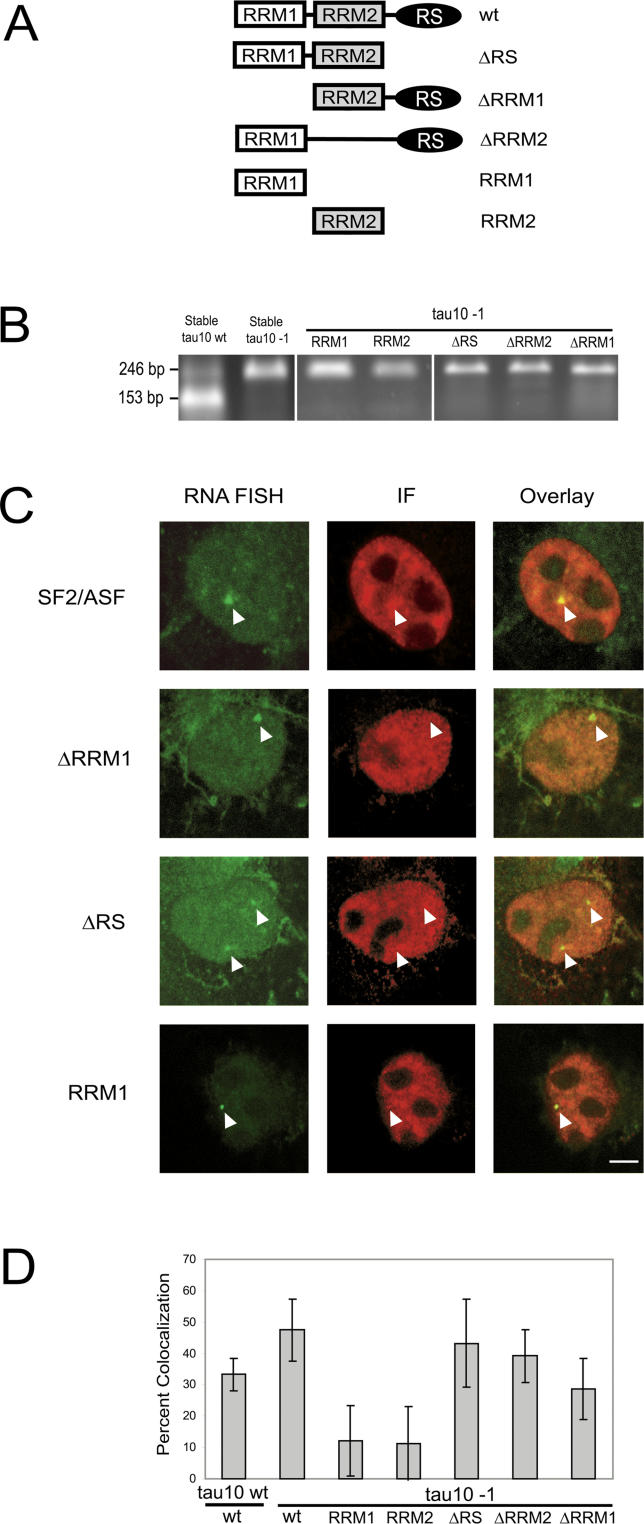
Deletion Mapping of Sf2/Asf Protein Domains Involved in Differential Recruitment (A) Schematic representation of T7-tagged mutants of SF2/ASF. (B) Semi-quantitative RT-PCR analysis of tau10−1 splicing upon expression of mutant SF2/ASF. Overexpression of the mutant proteins does not affect *tau* splicing. (C) Recruitment of splicing factors to *tau* transcription sites detected by combined RNA-FISH using specific probes against the *tau* minigene (green) and IF microscopy with anti-T7 antibody (red). Arrowheads indicate *tau* transcription sites. Scale bar = 2.5 μm. (D) Quantitation of percentage of cells with colocalization of *tau* RNA-FISH and splicing factor signals. Values represent averages from at least 50 transcription sites from three experiments ± SEM.

## Discussion

We have used quantitative single cell analysis to address the long-standing question of whether differentially spliced transcripts recruit distinct sets of basal pre-mRNA splicing factors. Biochemical methods have been unable to resolve this issue most likely due to the heterogeneity of isolated transcripts and spliceosomes and the dynamic nature of the spliceosome [2]. To circumvent these limitations, we have applied a validated microscopy recruitment assay allowing us to quantitatively analyze the association of splicing factors with alternatively spliced transcripts in vivo. Based on analysis of endogenous and exogenous SR proteins, we find that the accumulation of a subset of SR proteins at transcription sites correlates with the inclusion of a alternatively spliced exon. While all tested SR proteins accumulated at the transcription sites in cells where exon 10 of the *tau* gene is predominantly included, several SR proteins, including SF2/ASF, SC35, and SRp20, were found with reduced frequency at transcription sites in cells where tau exon 10 is excluded. The differentiation recruitment of a subset of splicing factors gives rise to combinatorial occupancy on the pre-mRNA.

Two general models for control of alternative splicing have been suggested. A first model envisions that alternative splicing events are controlled by specific alternative splicing factors. Clear examples of this model include *sex-lethal* in *Drosophila melanogaster* and *NOVA-1,* a human neuron specific alternative splicing factor [14,15]. However, use of dedicated alternative splicing regulators might be the exception to the rule. Considering that the majority of the approximately 25,000 human genes are alternatively spliced, many of them at multiple sites, it is hard to image the existence of specific splicing factors for all of these events. An alternative model suggests that the outcome of alternative splicing is largely determined by the combinatorial association of a commonly used set of splicing factors [8]. Evidence for this model is the fact that both in vivo and in vitro alternative splice site selection can be influenced by antagonistic titration of several splicing components [18–21]. In addition, this model is in line with the observation that several antagonistic splicing factors show tissue-specific abundance possibly contributing to tissue specific splicing patterns [34]. Our observations of differential recruitment of constitutive splicing factors verify the key prediction of this model that distinct sets of splicing factors associate with alternatively spliced transcripts. Furthermore, as expected for this model, recruitment of SF2/ASF involves the same protein regions as recruitment to a constitutively spliced transcript suggesting that recruitment to alternatively spliced introns is similar in its molecular mechanisms as recruitment to constitutive introns [27].

Given the differential recruitment of a subset of SR proteins, these factors might be expected to contribute to determining the splicing fate of *tau* exon 10. These proteins have indeed been shown to slightly favor inclusion of exon 10 in transient transfections in COS-7 cells [31]. However, in our hands only a moderate increase in exon 10 inclusion in the stably integrated *tau* minigenes was detected upon overexpression of SF2/ASF or SRp20. The limited ability of these differentially recruited proteins to shift the splicing outcome suggests that their mere presence at the template is not sufficient to control alternative splice site selection and that they are not the sole determinants of splice site choice, but that they cooperate with other spliceosome components. In the absence of information on the stoichiometry of SR proteins in spliceosomes involved in alternative splice site choice, it is difficult to predict the effect of their overexpression on a given template.

Our findings are in line with a dynamic, largely stochastic model for formation of the spliceosome on nascent RNA [8]. It seems likely that pre-mRNA splicing factors, which are known to roam the nucleus by diffusion [35,36], are temporarily captured near nascent pre-mRNAs by interaction with the C-terminal domain of the largest subunit of RNA polymerase [37,38]. Our observation of differential accumulation of SR proteins suggests that their putative interactions with the CTD are only transient, since stable interaction would result in equal recruitment of SR proteins to sites of transcription irrespective of splicing outcome. Since binding events are dynamic and probabilistic, not all assembled spliceosomes are identical and multiple types of spliceosomes containing different combinations of sets of splicing factors are formed. The nature of the formed spliceosomes is likely determined by the cellular and local abundance of factors, their post-translational modifications, and their interaction with specific regulatory splicing enhancers and repressor elements in the pre-mRNA. The various types of spliceosomes likely have distinct functional properties and recognize competing splice sites with different efficiencies, thus generating a mixed population of spliced transcripts. This model implies that alternative splice site choice is not absolute and that multiple isoforms of alternatively spliced transcripts should be generated for most transcripts. This is indeed frequently observed for many alternatively spliced pre-mRNAs, including endogenous *tau* [29,39–41]. A striking example of the stochastic, combinatorial nature of alternative splicing is the presence of different sets of isoforms of the axon guidance protein Dscam in *D. melanogaster*, where individual neurons express distinct repertoires of Dscam isoform combinations providing a potential mechanism to establish cell identity [42].

A role for combinatorial dynamics in the assembly of the spliceosome is fully consistent with the well-established dynamic changes the spliceosome undergoes during the splicing process where components are exchanged as the splicing reaction progresses [2]. The advantage of a combinatorial mechanism for spliceosome formation is that it offers the opportunity for cells to adjust splicing outcome rapidly in response to intracellular or extracellular signals without the need to synthesize a specific alternative splicing factor, but merely by modulating the binding of the existing splicing machinery by post-translational modifications or change in cellular distribution. This provides an efficient regulatory link that allows rapid response to physiological cues. Precedents for rapid, signal-mediated control of alternative splicing by these mechanisms exist. Signaling-mediated modulation of alternative splicing has been demonstrated for CD44 via phosphorylation of splicing factor Sam68 through the ERK pathway [43]. Similarly, splicing of an adenoviral E1A splicing reporter is affected by alterations of subcellular localization of hnRNPA1 controlled through the MKK(3/6)-p38 pathway [44]. Based on the sum of these considerations, we suggest that combinatorial recruitment of constitutive splicing factors is an essential property of spliceosomes that significantly contributes to generating protein diversity by alternative splicing and plays a critical role in regulation of splicing in vivo.

## Materials and Methods

### Generation of stable tau cell lines

To generate a plasmid suitable for generation of stable cell lines containing *tau* minigenes, the characterized tau10wt, −1, +3, and +14 sequences were modified by insertion of a zeocin selection cassette [30]. The *tau* minigene constructs in pSPL3b were first cut to completion with SphI and heat-deactivated. The entire digest was then end-filled using Klenow fragment and heat deactivated. The DNA was digested with AvrII to completion and the plasmid gel purified. The zeocin cassette was removed from pSV40 (Invitrogen, Carlsbad, California, United States) via double digest with NheI and PvuII and was ligated into the *tau* minigene plasmid in a 6:1 insert to vector ratio using T4 DNA ligase overnight at 16 °C. This yielded *pSPLZtau10*.

To generate stable cell lines COS-1 cells were grown on a 100-mm petri dish to 80% confluence in Dulbecco's modified essential medium (DMEM), 10% FBS, 0.3 mg/ml L-glutamine, and 0.1 mg/ml penicillin/streptomycin. Cells were trypsinized, resuspended in 10 ml of DMEM, and pelleted at low speed. The supernatant was removed and cells were resuspended in 200 μl of DMEM containing 4 μg of linearized *pSPLZtau10* plasmid, and 16 μg of salmon sperm DNA in a total volume of 30 μl. Cells were electroporated (2-mm gap cuvette, four pulses at 1-ms pulse, and 150-V) in a BTX electroporator ECM 830 (BTX, Holliston, Massachusetts, United States). Cells were placed on a 150-mm petri dish in DMEM overnight. For selection, cells were incubated in DMEM containing 0.9 mg/ml zeocin (Invitrogen). After 1–2 wk, single colonies were selected and expanded in DMEM containing 0.6 μg/ml zeocin. Clones were screened for expression of the *tau* minigene by RT-PCR using primers located in the *HIV-TAT* exons. Secondary screening was conducted by RNA-FISH. Stable lines were routinely grown in DMEM with 0.4 μg/ml zeocin.

### RT-PCR

Total RNA was extracted using either the RNAWhiz reagent (Ambion, Austin, Texas, United States) or the RNAqueous kit (Ambion) according to the manufacturer's instructions. Conventional RT-PCR was carried out as described in [45] except that Amplitaq Gold (Applied Biosystems, Foster City, California, United States). was used and the PCR reaction was initiated with a 10′ hot start at 95 °C. Quantitative real-time PCR, was performed using iQ SYBR Green Supermix (Biorad, Hercules, California, United States) following the manufacturer's instructions and products amplified, detected, and quantitated using a MyiQ (Biorad) single-color real-time PCR detection system on an iCycler (BioRad). The PCR routine consisted of 3 min at 94 °C, followed by 60 cycles of 20 s at 95 °C, and 1 min at 58 °C. For detection of *tau* mRNA containing exon 10, the following primers (forward, 5′-TGC AGA TAA TTA ATA AGA AGC TGG AT-3′; and reverse, 5,′- CCG GGA CGT GTT TGA TAT T-3′) located within exon 10 were used. This reaction yields an 81-nucleotide product. To measure the total amount of *tau* minigene mRNA, the following primers (forward, 5′-GTC ACC TGG ACA ACC TCA AA-3′; and reverse, 5′-CAG CTT GTC ACA GTG CAG TT-3′) located in the TAT donor exon upstream of exon 10 were used. This reaction yields a 62-nucleotide product. Amplification efficiencies were above 90% and production of single bands was confirmed by gel electrophoresis and melting curve analysis in the same run as measurements were done. RNA amounts were determined using calibrated standard curves using a dilution series over five orders of magnitude. Amounts of exon 10-containing mRNA were normalized to total *tau* minigene RNA. Values represent averages from at least two experiments ± standard deviations.

To determine the effect of overexpression of SR proteins on *tau* minigene splicing, COS-1 cells stably expressing tau10wt or tau10−1 were transiently transfected as described above with T7-tagged SR protein expression constructs in pCG-T7 as described in [32]. Microscopy was performed 16 h post-transfection.

### FISH and IF microscopy

Stable cell lines were treated for FISH exactly as described in [27] except that 0.1% Triton was used during the initial fixation instead of 0.5%. IF was performed exactly as described in [46]. For detection of endogenous splicing factors, FISH was carried out prior to IF without fixation or permeabilization steps between the two procedures. Cells transiently transfected with T7-tagged splicing factors were first subjected to IF, postfixed in 2% paraformaldehyde for 10 min followed by three rinses in PBS of 5 min each. RNA-FISH was then performed overnight. *Tau* was detected using probes against the entire minigene or a probe spanning the entire exon 10 generated by nick-translation as previously described [27].

### Microscopy and quantitative recruitment assay

Images were collected on a Leica TCS-SP confocal microscope. Single optical sections using 8× averaging were acquired by simultaneous scanning to avoid artifactual shift between the two optical channels. The 488-nm laser line of an Ar laser was used for detection of FITC RNA-FISH signals and the 568-nm line of a Kr laser for detection of TexasRed IF signals. Images were analyzed using Metamorph imaging software (Universal Imaging, Downington, Pennsylvania, United States). Recruitment was detected as accumulation of the IF signal at the *tau* site of transcription as previously described [27]. Positive recruitment was defined by two simultaneous criteria: a) the splicing factor signal was accumulated at the transcription site more than 2-fold above nucleoplasmic signal and b) at least half the splicing factor intensity peak overlapped with at least half of the RNA-FISH intensity peak. All quantitations were performed on unprocessed 8-bit grayscale images with no saturated pixels. At least 100 foci from at least three experiments were quantitated for each splicing factor per cell line. Statistical analysis was performed using a standard Student *t*-test.

For SF2/ASF mutants that do not give a distinct speckled pattern, quantitation was performed by measuring the average pixel intensity of the TexasRed signal at the transcription site and comparing it to the average TexasRed signal in the rest of the nucleus excluding nucleoli. Enrichment of the splicing factor signal of > 1.4-fold above its average nuclear intensity was defined as recruitment. This threshold was determined empirically by comparing the degree of recruitment of T-7 tagged SF2/ASF using the two quantitation methods. At least 50 foci from at least three experiments were analyzed for each SF2/ASF mutant.

## Abbreviations

DMEM: Dulbecco's modified essential medium
FISH: fluorescence in situ hybridization
snRNP: small nuclear ribonucleoprotein particles
wt: wild-type
